# Advertising and disclosure of funding on patient organisation websites: a cross-sectional survey

**DOI:** 10.1186/1471-2458-6-201

**Published:** 2006-08-03

**Authors:** Douglas E Ball, Klara Tisocki, Andrew Herxheimer

**Affiliations:** 1 Department of Pharmacy Practice, Faculty of Pharmacy, Kuwait University, Kuwait; 2 The UK Cochrane Centre, Oxford, UK

## Abstract

**Background:**

Patient organisations may be exposed to conflicts of interest and undue influence through pharmaceutical industry (Pharma) donations. We examined advertising and disclosure of financial support by pharmaceutical companies on the websites of major patient organisations.

**Method:**

Sixty-nine national and international patient organisations covering 10 disease states were identified using a defined Google search strategy. These were assessed for indicators of transparency, advertising, and disclosure of Pharma funding using an abstraction tool and inspection of annual reports. Data were analysed by simple tally, with medians calculated for financial data.

**Results:**

Patient organisations websites were clear about their identity, target audience and intention but only a third were clear on how they derived their funds. Only 4/69 websites stated advertising and conflict of interest policies. Advertising was generally absent. 54% of sites included an annual report, but financial reporting and disclosure of donors varied substantially. Corporate donations were itemised in only 7/37 reports and none gave enough information to show the proportion of funding from Pharma. 45% of organisations declared Pharma funding on their website but the annual reports named more Pharma donors than did the websites (median 6 vs. 1). One third of websites showed one or more company logos and/or had links to Pharma websites. Pharma companies' introductions were present on 10% of websites, some of them mentioning specific products. Two patient organisations had obvious close ties to Pharma.

**Conclusion:**

Patient organisation websites do not provide enough information for visitors to assess whether a conflict of interest with Pharma exists. While advertising of products is generally absent, display of logos and corporate advertisements is relatively common. Display of clear editorial and advertising policies and disclosure of the nature and degree of corporate donations is needed on patient organisations' websites. An ethical code to guide patient organisations and their staff members on how to collaborate with Pharma is also necessary, if patient organisations are to remain independent and truly represent the interests and views of patients. As many organizations rely on Pharma donations, self-regulation may not suffice and independent oversight bodies should take the lead in requiring this.

## Background

Patient advocacy and support organisations (hereafter 'patient organisations') are important in raising awareness of particular conditions, disseminating information about them, promoting the interests of patients and their carers and supporting them. Patients therefore tend to trust these organisations to act on their behalf in an unbiased manner [1]. However, relationships of patient organisations to pharmaceutical companies can compromise their independence (Table 1).

Since pharmaceutical companies (Pharma) and patient organisations have some interests in common, it is not surprising that funding relationships have developed between them [2]. However, at the same time, Pharma's competing and overriding responsibility to its shareholders based on ability to sell products may conflict with the best interests of patients [1,3,4]. Patient organisations offer Pharma two benefits: an avenue for influencing patients about prescription medicines which may not be advertised to the public (except notably in the USA and New Zealand); and a surrogate pressure group for influencing prescribers, policy makers and regulatory agencies on access to and use of their products. The industry has been accused of establishing its own 'patient organisations' in the past to suit its own agenda [3-5].

Recently the UK House of Commons Health Committee Enquiry into 'The Influence of the Pharmaceutical Industry' [6] noted that the level of support which patient organisations receive from Pharma is not known and that such groups need to openly declare "all significant funding and gifts in kind". Partly in response to these concerns, the Association of British Pharmaceutical Industry (ABPI) has revised its code of practice, now requiring all members to disclose "any involvement a pharmaceutical company has with a patient organisation". In particular, companies "must make public by means of information on their websites or in their annual report a list of patient organisations to which they provide financial support" [7,8]. Since they are not required to state the level or nature of the support, this 'disclosure' is thin. The government's response on disclosure by the patient organisations themselves was to leave this to self-regulation [9].

Others have also made the case for patient organisations to maintain their independence while still receiving and disclosing financial and other support from Pharma [1,4,10]. It is important for patients to know for themselves what interactions a particular patient organisation has with such companies so that they can judge whether it may be unduly influenced. The best approach in such cases is openness and disclosure. How much is disclosed has not been systematically studied although attention has been drawn to inadequacies. For example 20 of 67 Italian breast cancer patient groups had received funds from pharmaceutical companies but only 5 had conflict of interest statements [11].

To gauge the scope of the problem, we have examined disclosure of financial support and overt sponsorship, as well as advertising by companies on the websites of major national and international patient organisations.

## Methods

Websites for national and international patient organisations (patient organisations) based in the USA, UK, Australia, Canada, and South Africa were identified for ten major health conditions: cancer, heart disease, diabetes, asthma, cystic fibrosis, epilepsy, depression, Parkinson's disease, osteoporosis, rheumatoid arthritis – serious chronic conditions in which drug treatment is important.

Additional inclusion criteria were that the patient organisation:

• meet the membership criteria of the International Association of Patient Organizations (IAPO) (see below)

• be national or international (global) in nature (not local, regional or supranational e.g. European)

• be all-encompassing towards the condition of interest (not restricted to a particular form of the disease or a particular population group)

• be intended primarily for patients/carers in the country where the website was hosted (except for international patient organisations)

and that the website be in English and hosted in the USA, UK, Australia, Canada, or South Africa.

The membership criteria of the IAPO are that patient organisations should be non-profit and non-governmental, legally constituted, an international, regional, national or local organization, or an umbrella group; and demonstrate commitment to patients and be patient-driven (governed by patients, or have patients as most voting members or have a governance structure which solicits and is responsive to patient needs and views).

An assessment tool was developed for indicators of structural quality, general advertising, annual report availability, and pharmaceutical company funding, advertising or other involvement [see Additional file 1]. For structural quality indicators the assessment tool incorporated the Transparency and Accountability domains of the proposed 'Quality Criteria for Health Related Websites of the European Union' [12].

Websites were identified in the last week of December 2005 using Google [13] with the following search strategy:

"<***disease term***> international association OR society OR organisation OR organization OR foundation OR group OR support OR trust OR charity site:<***country domain***>"

The disease terms were cancer, heart, diabetes, asthma, epilepsy, depression, Parkinson's, "cystic fibrosis", osteoporosis and arthritis, and the country domains were org, uk, ca, au, za.

The first 50 hits of each search were examined by KT to identify potential eligible patient organisations. These were 'validated' by DB. DB and KT each evaluated the websites in January 2006 using the assessment tool. Differences were resolved through consensus, with AH as arbiter where opinions still differed.

### Advertisements

Banner advertisements, defined as 'third-party website advertisements which are an apparent source of income for the host organisation', were examined. We excluded charity shopping promotions which lead visitors to third-party online shopping or credit card sites where a percentage of spending is donated back to the patient organisation. We also excluded advertising of products from the organisation's merchandising arm.

### Financial summaries

Financial data on the net assets, total annual revenue (income) and income from membership dues and corporate donations were extracted from annual reports, excluding donations in kind and donations from foundations e.g. Amgen and GSK Foundations. Values were converted to US dollars using the interbank currency rate for 01/01/2006 on Oanda.com [14] [accessed Jan. 2006] without discounting or adjustment for inflation since these values were only being used as a rough measure of organisation size. Assets and revenue are reported to 2 significant figures. Where data were given the ratio of membership dues and corporate donations to annual revenue was taken to indicate the importance of these revenue sources to the organisation's work.

### Data analysis

Data were analysed using SPSS v13 (SPSS Inc., Chicago, USA) with Excel 2003 (Microsoft Inc., Redmond, USA) used for financial calculations. Simple tally and cross-tabulation was used for individual items, with calculation of medians for financial data. Since the study was descriptive and hypothesis-generating, formal statistical tests were not applied.

## Results

**Figure 1:**
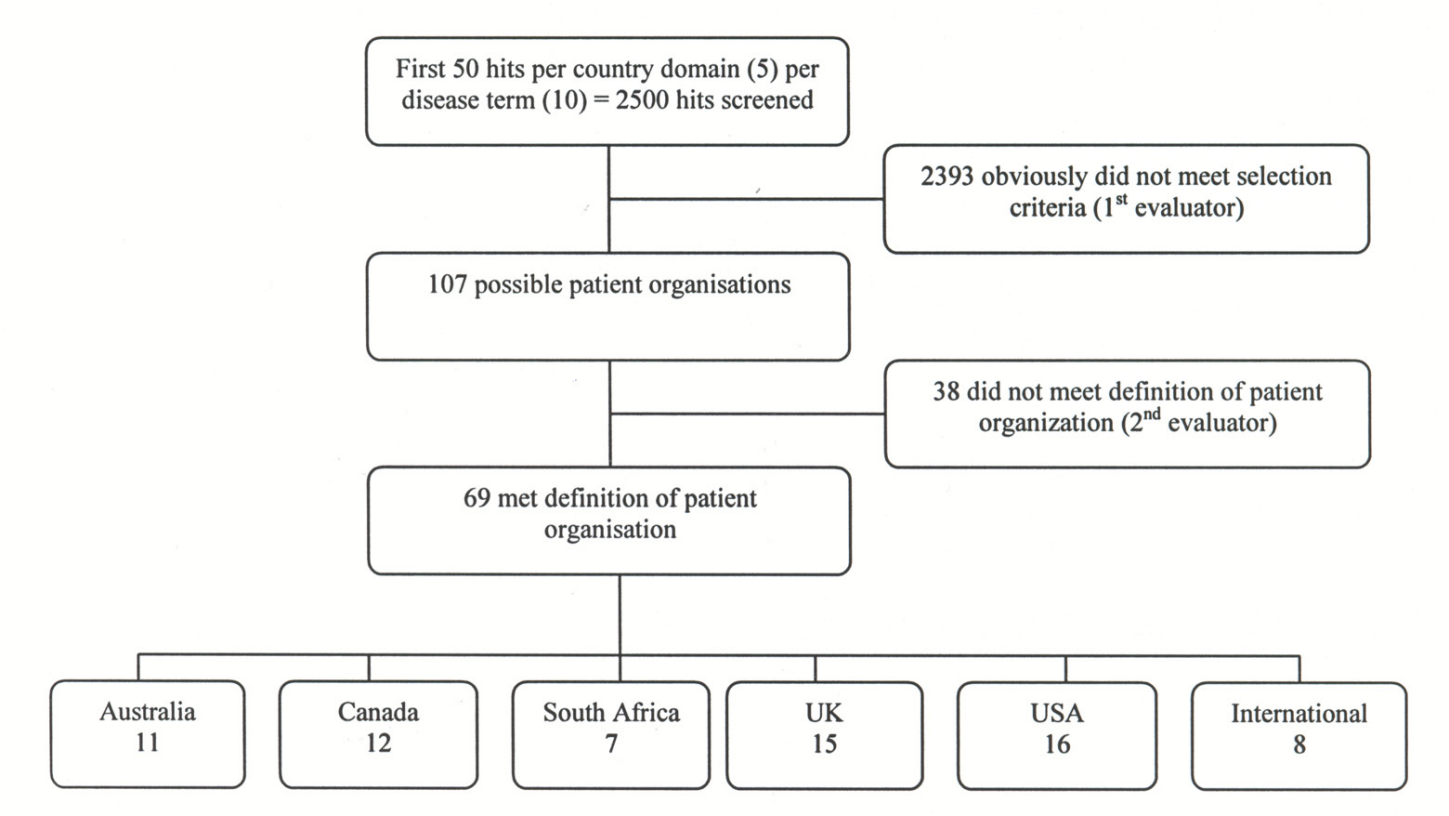
Flow diagram showing the selection process.

Of an initial 107 websites, 69 met our inclusion criteria (Figure 1). Table 2 lists the organisations and their URLs. The data are presented according to indicators of website quality, advertising, finances and disclosure of pharmaceutical company relationships.

### Structural quality indicators

Almost all the patient organisation websites (>96%) were transparent about who they were, e.g. had an 'About us' section, how to contact them and who the target audience was. They also displayed responsible partnering, i.e. links from their sites were mostly to reputable, non-profit governmental and non-governmental sites. Some had links to sponsoring organisations, including pharmaceutical companies (see later) and two depression websites linked to private psychiatry services. Few sites had explicit linking policies and, where these existed, they covered the rules for third parties to link to the patient organisation site rather than the reverse. Less than 20% stated an editorial policy describing how they derived and reviewed the health information, or clearly and regularly indicated the date of last update for their health-related content. Six of the 69 websites (4 from the USA) subscribed to the HONCode [15] or similar code for quality online health information (only four of these had a clear editorial policy). Transparency about funding was poor, with only a third of websites clear on how the organisation derived all of its funds (needed to indicate how funds are generated and which corporations give funds but not the amounts involved), although many solicited for donations and members, and/or offered online merchandise. No major differences were seen between countries although among South African groups a smaller proportion solicited user feedback about their website. In general South African patient organisation websites were less developed than their corresponding groups in the other countries.

### General advertising

Only 3/69 patient organisation websites carried banner advertising. One advertisement involved a pharmaceutical and we note it as an interesting example. The Arthritis Australia website carries a promotional banner for Pain Clear^®^, a branded generic version of paracetamol co-marketed by Arthritis New Zealand and a marketing company. A portion of the sale price goes to the patient organisation. We were not sure whether this was a 'true' banner advertisement since it resembles charity shopping.

**Figure 2:**
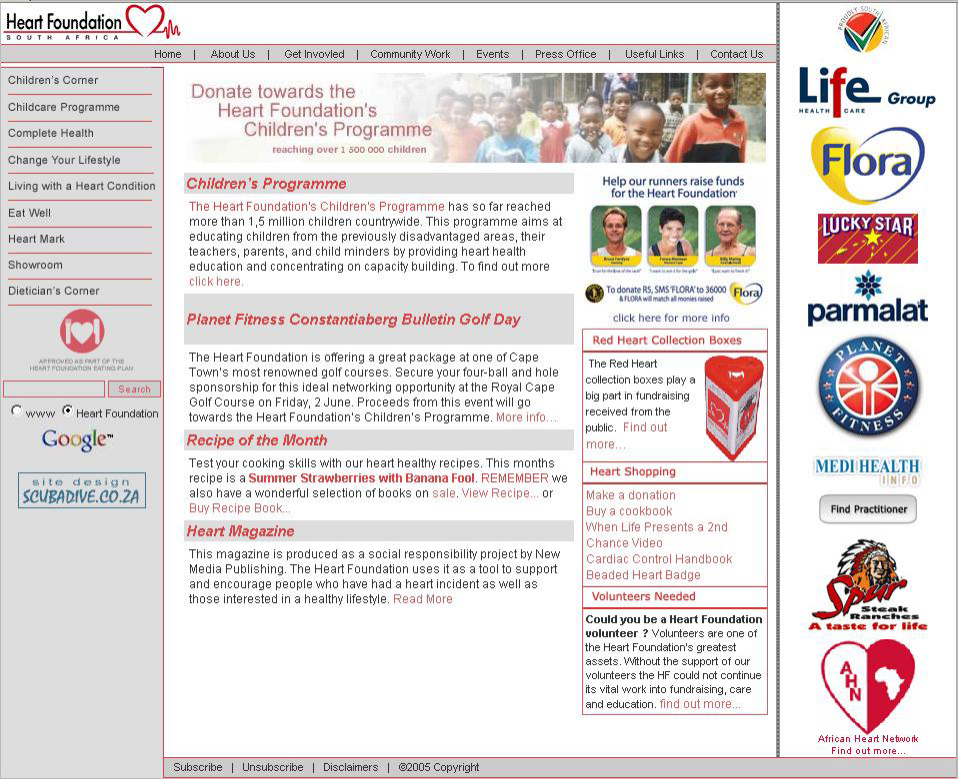
Screenshot of Heart Foundation of South Africa homepage with steakhouse sponsor logo.

We were also unsure about three instances where the patient organisation (two osteoporosis and one heart) carried what might be called 'banners' for their corporate sponsors on the homepage, linking to the company's website. These were described as a reward for the benefactor rather than advertisements but carry an obvious promotional advantage. A startling example was the Heart Foundation of South Africa website, sponsored by a local steakhouse (Figure 2).

Only 4/69 websites stated advertising policies (one in Australia, 2 in the UK and one in the USA), including two of those with banner advertisements. Four UK websites clearly stated that any health-related information on the website was independent from any funding which the organisation might receive.

### Annual reports and financial disclosure

Just over half (37; 54%) of sites had an 'up-to-date' annual or financial report on the website (range 2003–2005). Notably however, only 1 of 9 depression websites and 1 of 7 South African sites had an annual report (Table 3).

The mere presence of an annual report promises transparency and disclosure, but not all annual reports gave the same quality of information. Formats varied widely – some were little more than brief summaries of activities, while others were large documents with patients' stories and intended for soliciting donations. Acknowledgement of corporate sponsors varied greatly. Of the 37 reports, eight gave no information, 15 listed the names only (two only if the donation exceeded a certain threshold; some describing which programme the money supported), the Australian Heart Support report included a full page advertisement for Merck Sharpe & Dohme (MSD), one stated the donor's name and amount given (Epilepsy Action Australia) and 12 gave bands or tiers indicating the size of the donation, e.g. from $1,000 to $4,999. The number of tiers varied from two (American Cancer Society: $100,000 – $999,999 and $1,000,000 or more) to eight (covering donations from $10,000 through $5 million). The width and range of the tiers made it hard to estimate even the approximate value of donations. Further, these lists appeared to reflect only direct cash donations, excluding research grants and fellowships, e.g. the Heart and Stroke Foundation of Canada had 3 tiers for donations, but Pharma donations towards research fellowships were acknowledged separately with the company logo without mentioning the sum involved (a number of patient organisations administer research grants and fellowships, the money for which comes directly from Pharma).

The financial summaries in the annual reports also varied. Some organisations simply gave a gross figure for income while others broke it down using an assortment of categories to integrate corporate donations. In only 7/37 cases could the amount donated from corporate sponsors be identified. Pharma's contribution was not specified and whether sponsored research fellowships and scholarships were included was seldom clear. Examples of non-Pharma large corporate sponsors included computer manufacturers, banks, telecommunication companies and many local businesses. Non-profit trusts and foundations also made donations to most of the patient groups.

Using the annual reports, we tried to describe the financial status of the organisations and the contribution corporate donations and membership fees make to total income. The median net assets were $13 m (range $160,000 to $1,500 m) with median annual revenue (total income) of about $11 m (range $220,000 to $840 m). Cancer societies had by far the largest median assets ($630 m) and revenue ($260 m) (Table 3). Patient organisations in the USA had most assets and revenue, international organisations least (Table 4). Corporate donations made up 5% (Cancer Association of South Africa) to 65% (World Heart Federation) of annual income. Individual and corporate donations were about 70% of revenue on average. In our small sample, heart disease patient groups relied more on corporate donations than other disease societies.

Only two national organisations indicated the number of individual members – 165 for one, 170,000 for another. Associate members under umbrella patient organisations ranged from 12 to 193. Most preferred to 'measure' themselves by the use of their telephone helplines or website hits, but not all provided similar services to allow comparison on this basis. Membership fees were a line item in the financial summaries of 7 annual reports and contributed a median 7% of annual income (range 2 – 38 %). Organisation size in monetary terms seemed unrelated to indicators of transparency or relationships with Pharma companies (see below).

### Pharmaceutical company relationships and disclosure

Forty-five percent of 69 websites disclosed Pharma as a source of funding. However, we found discrepancies between the websites and the annual reports, with disclosure sometimes in one but not the other. Similar discrepancies were present for the number of Pharma donors disclosed: more were named in the annual reports than on the websites (median 6 vs. 1 respectively) (Table 3). National patient organisations tended to underrepresent Pharma companies on their websites while the opposite was true for international patient organisations (Table 4). We asked 28 of the 69 patient organisations which gave no indication of Pharma funding to clarify this; nine responded. Of these 4 had received Pharma donations, 5 said they had not.

Some annual reports mentioned a company repeatedly, e.g. donations from both Pfizer Arizona and Pfizer Arkansas, but this did not explain all the differences found. Seventeen percent of websites noted Pharma-sponsored events or programmes, which included research fellowships and educational initiatives. One presented a competition to win a Caribbean cruise associated with an analgesic medication (Arthritis Foundation, USA).

None of the websites clearly stated the proportion of income derived from Pharma. Eleven (16%) gave some indication of what the funding was used for, whether core operations, education or research. Only 13% noted that the funding was 'unrestricted', although in some additional cases, this was implied. One third of websites displayed Pharma logos and had links to Pharma websites. (Table 5). Some corporate introductions mentioned specific products likely to interest patients: for analgesia (arthritis), anti-allergy (asthma), nicotine replacement (cancer) and enzyme replacement (cystic fibrosis). No websites claimed to have been *established *by a pharmaceutical company. However, the World Parkinson's Disease Association website noted: "In 1990, Merck, Sharpe and Dohme International asked me to organize the first International Lay Associations Conference" [16], and one depression site (DepressioNet, Australia) claimed to owe its survival to Wyeth, which it repaid by giving the company patient stories and advice, participating in sales team and physician 'training', and with general promotional support: "Wyeth believe that it is important that their team who visit doctors and provide support materials etc (sic) have an understanding of the issues for 'people like us'. They seek to understand the needs of the end users of their products and to help our medical professionals to understand our needs (too)" [17].

## Discussion

Our findings show that national and international patient organisations are generally transparent on their websites about who they are, their intentions and target audience. However, many receive funding and possibly donations in kind from pharmaceutical companies (Pharma) and the extent of these relationships is incompletely acknowledged.

There are natural expectations that patient organisations and Pharma would have funding relationships [2,4] and surveys suggest that more than half of health advocacy groups receive money from Pharma [18,19]. However, their ultimate beneficiaries are different – patient members for the patient organisations, and shareholders for Pharma – and conflicts of interest may arise. Just as gifts (monetary or in kind) may induce feelings of loyalty and indebtedness in physicians which can influence medical decision-making [20,21] one may expect patient groups also to be caught between two masters, perhaps more so given the unequal nature of the relationship [4]. This is especially true for those that feel they could not function without financial help from industry [18] in the face of increasing demands on their resources [22].

The use of patient organisations as part of an overall strategy to influence authorities in the UK and EU has been 'exposed', [23] with recent examples. In 1999, Biogen actively recruited public opinion as part of its Action to Access campaign to coerce the National Health Service (NHS) in the UK to allow interferon beta be prescribed to multiple sclerosis patients, an illegal practice which the Medicines Control Agency then stopped [4]. In Denmark, local Pharma attempted to create a second migraine patient association when the existing group refused inappropriate "assistance" from the pharmaceutical industry [3]. A Dutch patient organisation for victims of inherited cardiovascular disorders which received half of its funds from Pharma, was criticised for defending a company (Pfizer) which had failed to inform doctors of a serious side-effect of its cholesterol-lowering drug [24]. More recently, while Roche has been largely out of the headlines during the clamour for wider prescribing of its breast cancer drug trastuzumab (Herceptin), reports suggest that they have been using patient groups to increase pressure on national and local authorities to widen prescribing [25]. These examples highlight how conflicts of interest can occur. Full disclosure is necessary to allow patients and other stakeholders to judge for themselves if they matter in particular circumstances [20].

Website structural quality indicators showed that the organisations were generally clear about themselves but not their funding. The lack of clear editorial policies and conflict of interest statements accords with the report that declaration of competing interests are rare in Italian breast cancer associations [11] and other international health advocacy groups [18], suggesting that the problem extends beyond disclosure on websites. Declaration of interests by medical advisers and office bearers of patient organisations is clearly also needed.

The websites were largely free from banner advertising, and those which did carry advertising did not carry Pharma advertisements. The absence of Pharma advertising is perhaps not surprising. Direct-to-consumer advertising of prescription medicines is not legal in any of the countries surveyed except the USA. In addition, national patient organisations are likely to have developed multiple sources of income and not want to give the impression of relying on Pharma advertising revenue. The failure to state an advertising policy on all but four of the websites meant that the official patient organisation position on advertising (on the website or in publications) was usually unknown.

One third of websites included 'advertisements' in the form of corporate introductions and/or display of the company's logo. Patient organisations may feel that they need to give something to the companies in return for their donation, but they should take care that such surreptitious advertising does not dent their independence or integrity. To help patient groups think of the issues involved and protect against conflicts of interest, we recommend that they develop and publish editorial and advertising policies, and administrators and medical advisors declare any Pharma-related interests.

Annual reports are often used to acknowledge and disclose sponsors by listing individual and corporate donors. However, a lack of information prevented assessment of the possible financial relations between Pharma and the patient groups. This recently emerged about the American Diabetic Association and National Osteoporosis Foundation in the USA [26]. In our survey, at one extreme, 65% of funding came from corporate donations suggesting that there are patient organisations which depend so heavily on this source that their independence could be threatened. Smaller organisations reliant on Pharma donations are more at risk and possibly more willing to push ethical boundaries in an effort to increase their advocacy power or to compete for donor attention [27]. These findings are consistent with previous accounts of the annual reports of national and international patient and health advocacy groups from North America, Europe and Australia, [18,22,28] and it is disappointing to note that there has been no improvement over the years. Standardised reporting of donors and donations, including specific listing of all Pharma benefactors, the amount or value of goods donated, and the proportion of income derived from Pharma should be implemented. The latter should be available on the website as well as stated in annual and financial reports.

The number of pharmaceutical companies providing donations is also an important factor in assessing to what influences an organisation is vulnerable [4], except in cases where the group has more of an advocacy role than direct patient care i.e. where it may be forwarding an agenda common to Pharma in general (as was seen with international patient organisations in this study). Disclosure was inadequate both on the websites and in the annual reports. Patient organisations should also be more transparent about their size and how far they represent patients. In this study, size varied a thousand-fold both in terms of financial indicators and membership. Most websites offered their services to non-members as well as members and preferred to describe their outreach in terms of number of website hits or telephone helpline calls, but these do not represent individual users. Ideally, both should be reported and governance structures described to indicate the role and voting rights of members.

Pharma may itself seek to establish patient groups sympathetic to its cause and products. Pharmaceutical companies played a part in establishing the International Association of Patient Organizations (IAPO) and the Global Alliance of Mental Illness Advocacy (GAMIAN); [4] when the Danish migraine association refused to accept funding Pharma tried to establish a rival migraine group [3]. The finding that Pharma had a role in the establishment of the World Parkinson's Disease Association was thus not unique. Another example is the establishment of an incontinence European Patient Association Network, which according to the director of the Continence Foundation UK followed "a meeting of patient groups arranged by Pfizer" [29]. One may wonder which other patient organisations groups owe their birth and life to Pharma.

The threat to the independence of patient organisations is real – in addition to accidental conflicts of interest, they may actually be targeted by Pharma. Some patient groups have been seen as mouthpieces for Pharma [21,25,26,30], addressing their lay members and regulators or insurance bodies, or unsuspecting partners in disease mongering [21,31]. They have a legitimate right to argue their case, but they can be taken seriously only if they are seen to be truly independent. It has been suggested that much of the funding for patient organisations comes from marketing rather than charity budgets, with funding from Merck and Pfizer to the Arthritis Foundation in the USA doubling when they launched their COX-2 inhibitors and falling back once safety concerns became evident [30]. Gifts to the organisations and their administrators can induce feelings of loyalty and dependency leading to compromised independence and modified practice as has been observed with doctors who receive gifts from Pharma [32]. Ethical codes guiding relationships between patient organisations and pharmaceutical companies along the lines of those for physicians would help to obviate potential conflicts of interest and assist smaller or younger groups maintain their independence.

Some patient organisations recognise that care and transparency is needed in interactions with Pharma [1,22,24]. Some openly declare their independence from Pharma funding, e.g. the Insulin Dependent Diabetes Trust [33]. In the UK, 50 charities have so far joined the ImPACT (Improving Accountability, Clarity and Transparency) Coalition under the National Council for Voluntary Organisations; they include about half a dozen patient organisations. However, the main thrust of the transparency is related to communicating to members and donors how funds are spent or invested, rather than how they are raised [34]. In Australia, the Consumers' Health Forum and Health Australia (the industry body in Australia) produced a joint guideline for cooperation between consumer organisations and Pharma [35]. But this suggests that sponsoring patient groups may increase the likelihood that a company's products might be listed on the national health reimbursement list – a clear industry bias [36]. Although relationships between patient organisations and Pharma have caused concern for some years [1,3,4,6,23], little has been done. Voluntary regulation has been suggested, not least by the UK government in response to the critical parliamentary report on the influence of the pharmaceutical industry in the UK [9]. This was supposed to begin by April 2006 but has not happened. The slow pace of reform, the difficulties of fundraising [18,22] combined with increasing numbers of charities wanting their share [22,37] and the tendency of some patient groups to push ethical boundaries 'for the cause' [27], suggest that self-regulation will be insufficient.

On the basis of our findings we recommend that:

□ National charity regulators/independent monitoring bodies should require standardised reporting by patient organisations of corporate donations in annual and financial reports and on their websites, including full disclosure of all Pharma donors and the nature and amounts of the donations;

□ National charity regulators/independent monitoring bodies should oblige patient organisations and other health charities with a web presence to maintain explicit editorial and advertising policies on their websites;

□ National charity regulators work with patient organisations to develop an ethical code of practice to guide relations with Pharma. Such an ethical code would give guidance on acceptance of donations from Pharma, what constitutes an acceptable gift to staff (travel, accommodation, food, etc.), and what, if any, are appropriate means of rewarding Pharma and other corporate donors;

□ Medical advisers of patient organisations should declare any possible conflicts of interest to the organisation's trustees and on the website.

Limitations of our study were:

The survey was restricted to major national and international patient organisations with websites. Although health informatics is an increasingly important means for patient groups to reach communities and their members, size and skills may prevent them maintaining a website. Organisations without a web presence were not represented and may differ from those included by being smaller and poorer. The focus on websites also ignored print or other material which patient organisations distribute and in which pharmaceutical companies may advertise. The fact that the study is a one-off cross-sectional survey of a rapidly changing medium of communication must also be borne in mind.

Patient organisations which focus more on advocacy than on actual patient care may also have been missed, as they may not maintain a website but rather directly approach their target audience, i.e. policymakers and regulators. If these are more influenced by Pharma, our sample could not show it. We also could not examine donations in kind or other forms of financial support since this not detailed on the websites or in the annual reports. Exclusion of Pharma Foundations may underrepresent the influence which Pharma has on patient organisations, e.g. both GlaxoSmithKline (GSK) and GlaxoSmithKline Foundation were listed in the top band (>$100,000) of donors to the National Parkinson Foundation.

## Conclusion

Patient organisations are clear about their purpose on their websites, but they do not disclose competing interests that they may have with pharmaceutical industry donors. While explicit advertising is generally absent, some patient organisations help companies by displaying logos and corporate advertisements. The lack of clear editorial and advertising policies makes this easier. Information about sponsors in annual reports often differs from that given on the website, and financial summaries rarely allow assessment of potential conflicts of interest. Greater guidance and disclosure is required if patient organisations are to remain independent and truly represent the views of patients.

## Competing interests

The authors are members of Health Action International Europe, a health advocacy organisation which works to increase access and improve the rational use of essential medicines.

## Authors' contributions

All authors contributed to the design of the study. DB and KT performed the online assessment of the websites with AH as arbiter. DB performed the data analysis and wrote the manuscript with contributions from the other authors. All authors critically reviewed the manuscript and approved the final version.

## Pre-publication history

The pre-publication history for this paper can be accessed here:



## Supplementary Material

Additional File 1 Appendix 1-Assessment tool for pharmaceutical sponsorship of patient organisations. The assessment tool used in the study. Click here for file

## Figures and Tables

**Table 1:** Relationships between pharmaceutical companies and patient organisations with potential for conflicts of interest or loss of independence

□ Monetary donations to the patient organisation
□ Payment of conference registration fees for patient organisation representatives
□ Payment for conference travel and accommodation for patient organisation representatives
□ Provision of non-educational gifts e.g. calendars, pens
□ Funding to hold local or international conferences
□ Funding of educational initiatives and meetings
□ Sponsoring research grants and study fellowships administered by the patient organisation for scientists in the field
□ Funding for core administrative functions
□ Donations in kind e.g. facilities, administration, training materials, books for libraries, website design
□ Provision of advocacy and educational material

**Table 2:** List of selected patient organisation websites.

**Condition**	**Organisation name**	**URL**	**Country**	**Date of access^1^**
**Arthritis**	Arthritis Australia		Australia	31 Dec. '05
	Arthritis Society		Canada	2 Jan. '06
	Arthritis Care		UK	2 Jan. '06
	National Rheumatoid Arthritis Society		UK	2 Jan. '06
	Arthritis Foundation		USA	2 Jan. '06
				
**Asthma**	Asthma Foundations of Australia		Australia	3 Jan. '06
	Asthma Society of Canada		Canada	3 Jan. '06
	Allergy/Asthma Information Association (AAIA)		Canada	3 Jan. '06
	Asthma UK		UK	3 Jan. '06
	British Lung Foundation		UK	3 Jan. '06
	Asthma and Allergy Foundation of America (AAFA)		USA	3 Jan. '06
	American Lung Association		USA	4 Jan. '06
				
**Cancer**	Cancer Council Australia		Australia	4 Jan. '06
	Canadian Cancer Society		Canada	4 Jan. '06
	Cancer Association of South Africa		S Africa	4 Jan. '06
	American Cancer Society		USA	4 Jan. '06
	Lance Armstrong Foundation		USA	4 Jan. '06
				
**Cystic fibrosis**	Cystic Fibrosis Australia		Australia	5 Jan. '06
	Canadian Cystic Fibrosis Foundation		Canada	5 Jan. '06
	Cystic Fibrosis Trust		UK	5 Jan. '06
	Cystic Fibrosis Foundation		USA	5 Jan. '06
	Cystic Fibrosis Worldwide		International	6 Jan. '06
				
**Diabetes**	Diabetes Australia		Australia	6 Jan. '06
	Canadian Diabetes Association		Canada	6 Jan. '06
	South African Diabetes Association		S Africa	6 Jan. '06
	Diabetes UK		UK	6 Jan. '06
	Diabetes Insight		UK	6 Jan. '06
	The Diabetes Research & Wellness Foundation		UK	6 Jan. '06
	American Diabetes Association		USA	6 Jan. '06
	International Diabetes Federation		International	9 Jan. '06
				
**Depression**	depressioNet		Australia	9 Jan. '06
	Mood Disorders Society of Canada		Canada	9 Jan. '06
	South African Depression and Anxiety group		S Africa	9 Jan. '06
	Depression Alliance		UK	9 Jan. '06
	SANE		UK	9 Jan. '06
	Depression and Related Affective Disorders Association (DRADA)		USA	9 Jan. '06
	Depression and Bipolar Support Alliance		USA	10 Jan. '06
	Freedom from Fear		USA	10 Jan. '06
	Trapped Minds		International	10 Jan. '06
	Befrienders Worldwide		International	10 Jan. '06
				
**Epilepsy**	Epilepsy Action Australia		Australia	10 Jan. '06
	Epilepsy Australia		Australia	10 Jan. '06
	Epilepsy Canada		Canada	10 Jan. '06
	Canadian Epilepsy Alliance		Canada	10 Jan. '06
	Epilepsy South Africa		S Africa	10 Jan. '06
	Epilepsy Action UK		UK	10 Jan. '06
	Epilepsy Foundation		USA	10 Jan. '06
	International Bureau for Epilepsy (IBE)		International	10 Jan. '06
				
**Heart disease**	National Heart Foundation Australia		Australia	10 Jan. '06
	Heart Support – Australia Ltd (HSA)		Australia	10 Jan. '06
	Heart and Stroke Foundation		Canada	10 Jan. '06
	Heart Foundation of South Africa		S Africa	10 Jan. '06
	British Heart Foundation		UK	11 Jan. '06
	Heart UK		UK	11 Jan. '06
	American Heart Association		USA	11 Jan. '06
	World Heart Federation		International	11 Jan. '06
				
**Osteoporosis**	Osteoporosis Australia		Australia	11 Jan. '06
	Osteoporosis Canada		Canada	11 Jan. '06
	National Osteoporosis Foundation of South Africa		S Africa	11 Jan. '06
	National Osteoporosis Society		UK	11 Jan. '06
	National Osteoporosis Foundation		USA	11 Jan. '06
	International Osteoporosis Foundation		International	11 Jan. '06
				
**Parkinson's disease**	Parkinson Society Canada		Canada	11 Jan. '06
	Parkinson Association South Africa		S Africa	11 Jan. '06
	Parkinson's Disease Society		UK	11 Jan. '06
	National Parkinsons Foundation		USA	11 Jan. '06
	American Parkinson Disease Association, Inc.		USA	11 Jan. '06
	Parkinson's Action Network		USA	11 Jan. '06
	World Parkinson Disease Association		International	11 Jan. '06

^1^This is the date of first access. Website evaluators did not necessarily visit the site on the same day. Many sites were revisited to resolve differences between assessors.

**Table 3:** Financial indicators and number of Pharma donors from annual reports and websites, analysed by disease.

**Disease group & no. of patient organisation websites**	**Annual report available from website**	**Median Net assets ($000,000s) [n]^b^**	**Median annual revenue^c ^($000,000s) [n]**	**Donations^d ^received as proportion of revenue; median [n]**	**Corporate donations^e ^as prop. of revenue; median [n]**	**Median no. (range) Pharma sponsors (Website)^f^**	**Median no. (range) Pharma sponsors (Ann. report)**
**Arthritis (5)**	4	5.2 [4]	5.3 [3]	0.52 [3]	0.07 [1]	8.5 (5–10)	3.5 (0–19)
**Asthma (7)**	4	4.7 [4]	5.0 [4]	0.65 [4]	0.32 [2]	4 (0–14)	6.5 (0–9)
**Cancer (5)**	4	630.0 [3]	260.0 [4]	0.58 [3]	0.05 [1]	0 (0–2)	3 (0–8)
**Cystic fibrosis (5)**	4	1.0 [3]	6.3 [3]	0.55 [3]	-	0 (0–2)	2.5 (0–10)
**Diabetes (8)**	4	20.7 [3]	98.0 [3]	0.77 [3]	-	2 (0–15)	14.5 (1–29)
**Depression (9)**	1	1.1 [1]	3.0 [1]	0.92 [1]	-	0	11
**Epilepsy (8)**	4	1.9 [3]	4.5 [4]	0.58 [4]	-	0.5 (0–6)	3.5 (0–8)
**Heart disease (8)**	5	47.0 [3]	140.0 [4]	0.65 [4]	0.65 [1]	2 (0–7)	1 (0–38)
**Osteoporosis (6)**	4	7.2 [3]	4.9 [4]	0.66 [4]	0.38 [1]	3.5 (0–21)	11 (6–13)
**Parkinson's (7)**	3	22.0 [2]	9.5 [3]	0.78 [3]	0.07 [1]	1 (0–11)	7 (0–17)

**Total (n = 69)**	37	13.0 [29]	11.0 [33]	0.68 [32]	0.11 [7]	1 (0–21)	6 (0–38)

**Notes**:

^a^See Methods for description of data standardisation across currency and year.

^b^Not all annual reports contained the necessary information; n = number of valid data entries.

^c^Total income over the year of the annual report from all sources (excluding costs of fundraising activities).

^d^Donations includes legacies/bequests and membership dues, but excludes donated services and materials and special events.

^e^Corporate donations generally exclude donated services and materials, sponsored fellowships and research awards.

^f^Only those websites having an annual report for comparison

**Table 4:** Financial indicators and number of Pharma donors from annual reports and websites, analysed by country.

**Country & no. of patient organisation websites**	**Annual report available from website**	**Median net assets ($000,000s) [n]^b^**	**Median annual revenue^c ^($000,000s) [n]**	**Donations^d ^received as proportion of revenue; median [n]**	**Corporate donations^e ^as prop. of revenue; median [n]**	**Median no. (range) Pharma sponsors (Website)^f^**	**Median no. (range) Pharma sponsors (Ann.report)**
**Australia (11)**	4	12.0 [2]	51.0 [4]	0.79 [4]	-	0 (0–9)	3 (1–7)
**Canada (12)**	6	-	5.7 [3]	0.70 [3]	0.22 [2]	1.5 (0–7)	7.5 (0–24)
**S. Africa (7)**	1	16.0 [1]	8.8 [3]	0.10 [1]	0.05 [1]	0	0
**UK (15)**	9	8.0 [9]	17.0 [8]	0.64 [8]	0.11 [1]	1 (0–8)	4 (0–9)
**USA (16)**	13	44.0 [13]	24.0 [13]	0.65 [12]	0.30 [2]	0 (0–15)	10 (0–38)
**Global (8)**	4	1.3 [4]	1.3 [4]	0.69 [4]	0.65 [1]	4.5 (0–21)	0.5 (0–13)

**Total (69) [%]**	37 [54%]	13.0 [29]	11.0 [33]	0.68 [32]	0.11 [7]	1 (0–21)	6 (0–38)

**Notes**:

^a^See Methods for description of data standardisation across currency and year.

^b^Not all annual reports contained the necessary information; n = number of valid data entries.

^c^Total income over the year of the annual report from all sources (excluding costs of fundraising activities).

^d^Donations includes legacies/bequests and membership dues, but excludes donated services and materials and special events.

^e^Corporate donations generally exclude donated services and materials, sponsored fellowships and research awards.

^f^Only those websites having an annual report, for comparison.

**Table 5:** Indicators of interactions with pharmaceutical companies analysed by country.

**Country & no. of patient organisation websites**	**Proportion of income from Pharma is clear**	**Use of Pharma funding is clear**	**Clear that Pharma funding is 'unrestricted'**	**Pharma logos are present**	**Pharma introductions present**	**Evidence of Pharma sponsored events, etc**.	**Links to Pharma websites present**
**Australia (n = 11)**	0	0	2	4	1	1	3
**Canada (n = 12)**	0	2	1	7	3	2	6
**S. Africa (n = 7)**	0	0	0	3	0	0	2
**UK (n = 15)**	0	3	2	5	1	2	4
**USA (n = 16)**	0	5	3	2	2	5	5
**Global (n = 8)**	0	1	1	2	0	2	1

**Total (n = 69) n [%]**	1 [1%]	11 [16%]	9 [13%]	23 [33%]	7 [10%]	12 [17%]	21 [30%]

**Key**: Pharma – pharmaceutical companies or industry
