# Supplementary material for: Advertising and disclosure of funding on patient organisation websites: a cross-sectional survey
Source: BMC Public Health. 2006 Aug 3;6:201. doi: 10.1186/1471-2458-6-201 (PMC1557495; doi:10.1186/1471-2458-6-201)
Supplement: Additional File 1 — Appendix 1-Assessment tool for pharmaceutical sponsorship of patient organisations. The assessment tool used in the study. [file 1471-2458-6-201-S1.doc]

**Appendix 1 –Assessment tool for pharmaceutical sponsorship of patient organisations.**

Website URL: ________________________________ Date: __________________

| **Criterion** | **Yes** | **No** | **Comments** |
| --- | --- | --- | --- |
| **Transparency and honesty** |  |  |  |
| 1. Transparency of **provider** of site – including name, physical address and electronic address of the person or organisation responsible for the site |  |  |  |
| 1. Transparency of **purpose** and objective of the site |  |  |  |
| 1. **Target audience** clearly defined (Further detail on purpose, multiple audience could be defined at different levels) |  |  |  |
| 1. Transparency of all sources of **funding** for site (grants, sponsors, advertisers, non-profit, voluntary assistance |  |  |  |
|  |  |  |  |
| **Accountability** |  |  |  |
| 1. Accountability - **user feedback**, and appropriate oversight responsibility (such as a named quality compliance officer for each site) |  |  |  |
| 1. Responsible **partnering** – all efforts should be made to ensure that partnering or linking to other websites is undertaken only with trustworthy individuals and organisations who themselves comply with relevant codes of food practice |  |  |  |
| 1. **Editorial policy** – clear statement describing what procedure was used for selection of content |  |  |  |
|  |  |  |  |
| **Updating** |  |  |  |
| 1. Clear and regular updating of the site, with date of up-date clearly displayed for each age and/or item as relevant. Regular checking of relevance of information. |  |  |  |
| 1. Date of last update clearly indicated on homepage |  |  |  |
| *If present*, was last updated within the last year |  |  |  |
|  |  |  |  |
| **Source of funding and advertising policy** |  |  |  |
| 1. The site displays banner advertising which is apparently a source of income |  |  |  |
| 1. A page provides a description of the site’s advertising policy (including that it does not accept advertising if appropriate) |  |  |  |
| 1. Separation between editorial content and advertising/funding is clearly stated |  |  |  |
| 1. Presence of annual/financial report |  |  |  |
| 1. Date of last annual/financial report |  |  |  |
| 1. Total budget and total income from donations (state currency) |  |  |  |
| 1. Total number of individual [and corporate] members |  |  |  |
|  |  |  |  |

| **Pharmaceutical company funding** |  |  |  |
| --- | --- | --- | --- |
| 1. Pharmaceutical companies are identified as providing funds to the association |  |  |  |
| 1. No. of pharmaceutical company sponsors |  |  |  |
| 1. The proportion of income derived from pharmaceutical companies is [clearly] indicated |  |  |  |
| 1. Clear statement that pharmaceutical company funding is “unrestricted” |  |  |  |
| 1. Use of pharmaceutical company funding is clearly indicated |  |  |  |
| *If indicated*, it specifies whether for core, educational, or research purposes | **Core** | **Educ.** | **Research** |
| 1. Date of last update on page which provides detail of donations/sponsorship |  |  |  |
| *If present*, last update was within last 12 months |  |  |  |
| 1. Clear statement that organisation was founded by a pharmaceutical company |  |  |  |
|  |  |  |  |
| **Pharmaceutical company advertisements and sponsored events** |  |  |  |
| 1. Pharmaceutical company banner advertisements for products are present |  |  |  |
| 1. Pharmaceutical logos are used anywhere in the website e.g. when naming sponsors |  |  |  |
| 1. Pharmaceutical company corporate advertisements or ‘introductions’ are present |  |  |  |
| 1. There are more pharmaceutical company-related advertisements that those from other sources |  |  |  |
| 1. There is evidence of pharmaceutical company-sponsored education programs, courses, information or events |  |  |  |
| 1. There are links to pharmaceutical company or pharmaceutical company-sponsored websites |  |  |  |

**General comments:**
